# Determinants of human papillomavirus 16 serological conversion and persistence in a population-based cohort of 10 000 women in Costa Rica

**DOI:** 10.1038/sj.bjc.6602088

**Published:** 2004-08-03

**Authors:** S S Wang, M Schiffman, R Herrero, J Carreon, A Hildesheim, A C Rodriguez, M C Bratti, M E Sherman, J Morales, D Guillen, M Alfaro, B Clayman, R D Burk, R P Viscidi

**Affiliations:** 1 Division of Cancer Epidemiology and Genetics, National Cancer Institute, Bethesda, MD 20892-7234, USA; 2 Proyecto Epidemiologico Guanacaste, 301-6151, San Jose, Costa Rica; 3 Stanley Division of Developmental Neurovirology, Department of Pediatrics, Johns Hopkins University School of Medicine, Baltimore, MD 21205, USA; 4 Albert Einstein College of Medicine, Bronx, NY 10461, USA

**Keywords:** HPV, serology, persistence, conversion, cervix, cancer

## Abstract

Determinants of human papillomavirus (HPV)-16 serological conversion and persistence were assessed in a population-based cohort of 10 049 women in Guanacaste, Costa Rica. Serologic responses to HPV-16 were measured in 7986 women by VLP-based enzyme-linked immunosorbent assay at both study enrolment (1993/94) and at 5–7 years of follow-up. Seropositive women were defined as ⩾5 standard deviations above the mean optical density obtained for studied virgins at enrolment (*n*=573). Seroconnversion (*n*=409), persistence (*n*=675), and clearance (*n*=541) were defined based on enrolment and follow-up serology measurements. Age-specific distributions revealed that HPV-16 seroconversion was highest among 18- to 24-year-old women, steadily declining with age; HPV-16 seropersistence was lowest in women 65+ years. In age-adjusted multivariate logistic regression models, a 10-fold risk increase for HPV-16 seroconversion was associated with HPV-16 DNA detection at enrolment and follow-up; two-fold risk of seroconversion to HPV-16 was associated with increased numbers of lifetime and recent sexual partners and smoking status. Determinants of HPV-16 seropersistence included a 1.5-fold risk increase associated with having one sexual partner during follow-up, former oral contraceptive use, and a 3-fold risk increase associated with HPV-16 DNA detection at both enrolment and follow-up. Higher HPV-16 viral load at enrolment was associated with seroconversion, and higher antibody titres at enrolment were associated with seropersistence.

Most genital human papillomavirus (HPV) infections are transient: within 2 years of incident infection, HPV DNA becomes undetectable in approximately 90% of women (Ho *et al*, 1995). Moreover, not all women infected with HPV seroconvert; only about half of all HPV DNA-positive women test positive for corresponding type-specific antibodies using available assays (Kirnbauer *et al*, 1994; Le Cann *et al*, 1995). For those women who have seroconverted, however, detection of serum antibodies to HPV capsids is a valid marker of current and past type-specific HPV exposure (Wideroff *et al*, 1995, 1999; Carter *et al*, 1996; Sasagawa *et al*, 1998; Touze *et al*, 2001).

We previously reported population-based seroprevalence of HPV-16, -18, -31, and -45 in our 10 000 women population-based study in Costa Rica at enrolment; we confirmed the waning detection of HPV antibodies with age and the determinants of seroprevalence to include increasing lifetime number of sexual partners and smoking (Wang *et al*, 2003), as similarly reported in other prevalence studies (Stone *et al*, 2002; Nonnenmacher *et al*, 2003).

To extend our cross-sectional findings, we have now completed HPV-16 serology measurements at a second time point, after 5–7 years of follow-up. Human papillomavirus 16 was selected because it accounts for the majority of cervical cancers worldwide (Munoz *et al*, 2003) and is the focus of immunology and vaccinology research (Lowy and Frazer, 2003). To further our understanding of HPV serology in the natural history of cervical cancer, we report here the epidemiologic characteristics and determinants of HPV-16 serologic conversion and persistence in our Guanacaste, Costa Rica cohort.

## METHODS

### Study population

This study was conducted in the population-based cohort of 10 049 women in Guanacaste, Costa Rica. As previously described, study enrolment was conducted in 1993–94 with approval from the NCI and local institutional review boards (Herrero *et al*, 1997; Hildesheim *et al*, 2001; Bratti *et al*, 2004). Briefly, the cohort was a representative sample of the adult female population in Guanacaste, Costa Rica in 1993–94. Of 10 738 women eligible for the study, 10 049 were interviewed (94%); refusals were women who did not show up for their appointment despite multiple invitations. At enrolment, study participants completed a risk factor questionnaire that addressed demographic characteristics and behavioural and reproductive practices. Follow-up of the cohort was conducted for 5–7 years until cohort exit, which began in 2000. For the present analysis, follow-up data were available for 8084 women; the median length of follow-up was 6.4 years. In the present analysis, a histologic diagnosis of CIN3 or cancer during follow-up (*n*=75) was considered the reference standard of serious, newly diagnosed high-grade disease. Plasma specimens collected from women at enrolment and at the end of follow-up (concurrently at the time of diagnosis for cases) were used for measurement of serologic responses to HPV-16. Of the 10 049 women enrolled in the cohort, serology results from enrolment specimens were obtained for 9951 women. Of the 8084 women selected for follow-up (women with hysterectomies or prevalent high-grade neoplasia were not followed) (Bratti *et al*, 2004), plasma samples were obtained from 8058 women. However, of the 8058 women with plasma at follow-up, 72 women did not have plasma at enrolment. Our final analytic population thus included 7986 women for whom plasma was available at both time points.

### Serological measurements

Plasma samples collected at study enrolment and at the end of follow-up were tested for anti-HPV L1 antibodies (IgG) at the Johns Hopkins Medical Institutions by a VLP-based enzyme-linked immunosorbent assay (ELISA) for HPV-16. Serology measurements were conducted as previously described (Wang *et al*, 2003). Briefly, each batch comprised approximately 2000–3000 specimens, including intra- and interbatch reliability repeat specimens and random samples of 200 virgins in the study population, which permitted the calculation of batch-specific serology cut-points (see below).

### Human papillomavirus DNA testing

Cervical cytologic specimens collected using a Dacron swab at enrolment and follow-up were tested for HPV DNA using L1 MY09/MY11 consensus primer methods, with TaqGold polymerase and dot-blot typing, as previously described (Herrero *et al*, 2000). The results for HPV-16 testing were available for 7367 women included in the present analysis, because virgins did not undergo pelvic examinations at enrolment.

### Statistical methods

Consistent with our previous analysis, serology results were dichotomised as antibody positive or negative (Wang *et al*, 2003). Women whose mean optical density (OD) measurements were five standard deviations above that obtained for the concurrently tested virgins (minus outliers in the virgin OD distribution) were categorised as seropositive. All other women below the five standard deviation cut-point were defined as seronegative. This definition of seronegative women included, at enrolment, 3110 women above and 5307 women below one standard deviation of study virgins; at follow-up, this definition included 1470 women above and 5495 women below one standard deviation of study virgins. The cutoff was calculated independently for each test batch, by comparison to the distribution of the values obtained for the concurrently tested virgins in that batch (*n*=200). To demonstrate that our results were robust, we also compared seropositive women to seronegative women, defined as less than one standard deviation from studied virgins.

#### Defining serologic outcomes

The enrolment population was first dichotomised as HPV-16 seropositive (*n*=1534) and seronegative (*n*=8417). Of the 1216 (79%) HPV-16 seropositive women with follow-up data, 675 (55.5%) remained seropositive at follow-up and 541 (44.5%) tested seronegative (Table 1

**Table 1:** Definition of study outcomes: HPV-16 seroconversion, seroclearance, and seropersistence

	**Serology measurement**		
	**Enrolment**	**Follow-up**	**Enrolment and follow-up**
**HPV-16 serological status**	**(*n*=9951)**	**(*n*=8058)**	**(*n*=7986)**
Negative	−	−	6361
Clearance	+	−	541
Conversion	−	+	409
Persistence	+	+	675

). Of the 6770 (80%) HPV-16 seronegative women at enrolment with follow-up data, 409 (6%) seroconverted and 6361 (94%) remained seronegative (Table 1). We identified determinants of seroconversion by comparing the 409 women who seroconverted to the 6361 who remained seronegative. Likewise, we identified determinants of seropersistence by comparing the 675 women who remained seropositive at follow-up to the 541 who became seronegative.

Age-specific distributions of seroconversion, serologic persistence, and serologic clearance were graphically compared with age category in years (18–24, 25–29, 30–44, 45–64, 65+ years). Age was calculated as the woman's age at the midpoint of her follow-up.

#### Determinants of serologic status

All HPV cofactors reported previously in Costa Rica (Hildesheim *et al*, 2001) were thoroughly investigated with regard to serologic outcomes. Specifically, univariate associations for both outcomes (seroconversion and seropersistence) were assessed for the following behavioural and reproductive variables assessed at enrolment and follow-up via questionnaire: number of sexual partners (lifetime at enrolment, and recent, defined as during the follow-up period and thus includes both previous and new partners), use of oral contraceptives (OCs) (never, former, current), smoking status (never, former, current), early age at first intercourse (defined as <16 years), number of live births and number of pregnancies (lifetime at enrolment and recent, defined as during the follow-up period), years between menarche and sexual debut, and self-reported sexually transmitted diseases. For smoking and OC use, a combined variable was constructed from enrolment and follow-up data (e.g. enrolment/follow-up – never/never, former/former, current/current, never/current, current/former, former/current). Odds ratio estimates with 95% confidence intervals (CIs) were obtained to assess the magnitude and statistical significance of the associations between these variables and HPV serostatus. Presence of HPV-16 DNA at enrolment and follow-up was also assessed considering all combinations for DNA status (e.g. enrolment/follow-up – negative/negative, negative/positive, positive/negative, positive/positive).

We assessed HPV-16 semiquantitative viral load and the OD for serological measurement at enrolment as potential determinants of HPV seroconversion and seropersistence. Specifically, we assessed whether women with higher OD measurements at enrolment were more likely to remain seropositive at follow-up, and whether higher HPV-16 viral load at enrolment, as measured by signal strength from PCR, was associated with seroconversion or seropersistence.

Variables independently and statistically significantly associated with seroconversion or seropersistence in our univariate analyses were included in our final multivariate models; they included number of lifetime sexual partners reported at enrolment (1, 2–3, 4+), number of sexual partners during follow-up (0, 1, 2+), smoking (never/never, never/current, current/current, current/former, former/former, former/current), OC use (never/never, never/current, current/current, current/former, former/former, former/current), and HPV-16 DNA status (positive/positive, positive/negative, negative/positive, negative/negative) as measured by PCR. Our final model also adjusted for age at the midpoint of the woman's follow-up (years), and serology batch. We excluded women who remained virgins throughout the duration of study follow-up (*n*=273).

Finally, the age-adjusted association between HPV-16 DNA and serological status among the 75 women with a CIN3/cancer diagnosis during study follow-up was assessed (odds ratio and 95% CI). For these cases, the follow-up testing was carried out at the time of diagnosis before treatment. CIN3/cancers diagnosed at enrolment were censored for follow-up and did not have a second time point for which serology measurements were made; thus, they were excluded from all analyses. Statistical analyses were conducted on SAS 8.2 for Windows and STATA 7.0.

## RESULTS

**Figure 1:**
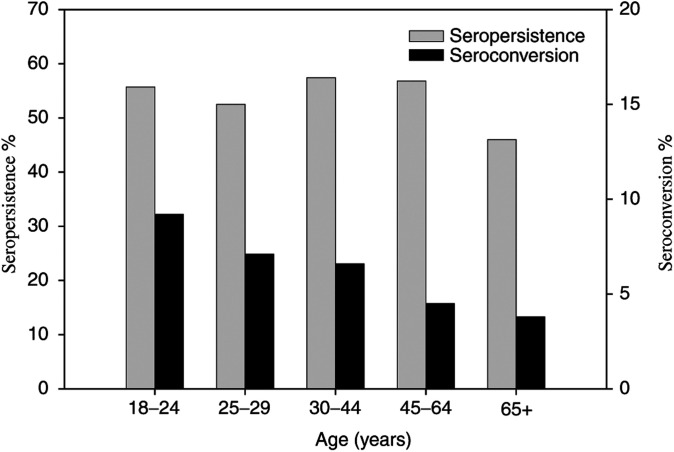
Age distribution of HPV-16 seroconversion and persistence in Guanacaste, Costa Rica women at midpoint age (years) during follow-up.

Seroprevalence of HPV-16 at both cross-sectional time points (enrolment in 1993–94, and follow-up in 2000) was 15%. In all, 55% (675 of 1216) of women seropositive at enrolment remained seropositive for HPV-16 at follow-up, while 6% (409 of 6770) of initially HPV-16 seronegative women were seropositive at the time of follow-up. As shown in Figure 1

, serologic status varied by age. Of women seronegative at enrolment, seroconversion was highest in women 18–24 years old and steadily declined with increasing age. Of women seropositive at enrolment, HPV-16 serologic persistence was above 50% for all age groups except in women 65 years and older.

### Determinants of HPV-16 seroconversion

Univariate analyses for determinants of HPV-16 seroconversion, compared to women who remained seronegative, revealed a number of potential determinants, which were subsequently included in the final multivariate logistic regression model. After adjustment for age and serology batch, and excluding women who remained virgins for the duration of the cohort study, two or more lifetime number of sexual partners and one or more sexual partner (not necessarily new partners) during study follow-up were statistically significantly associated with an increased risk for seroconversion (Table 2

**Table 2:** Final logistic regression model demonstrating the association between recent and lifetime number of sexual partners, smoking status, and HPV-16 DNA status with HPV-16 seroconversion in the Guanacaste Study (*n*=6587), adjusting for age and serology batch, and excluding virgins

**Determinants of HPV-16 seroconversion**	**HPV-16 seroconversion (*n*=400)^a^ (no. (%))**	**Remained seronegative (*n*=6128)^a^ (no. (%))**	**OR (95% CI)**
*Lifetime number of sexual partners at enrolment*			
0–1 (referent)	17 (4.5)	3707 (95.5)	1.0
2–3	144 (8.7)	1512 (91.3)	1.9 (1.5–2.4)
4+	39 (11.0)	317 (89.0)	1.9 (1.3–2.9)
			
*Number of sexual partners during follow-up*			
0 (referent)	219 (4.9)	4282 (95.1)	1.0
1	82 (8.4)	891 (91.6)	1.5 (1.1–2.0)
2+	56 (13.5)	358 (86.5)	2.2 (1.6–3.1)
			
*Smoking*			
Never/never (referent)	341 (5.9)	5437 (94.1)	1.0
Never/current	6 (8.6)	64 (91.4)	1.1 (0.5–2.6)
Current/current	11 (6.0)	171 (94.0)	0.7 (0.4–1.4)
Current/former	20 (16.8)	99 (83.2)	2.8 (1.7–4.8)
Former/former	11 (3.9)	273 (96.1)	0.7 (0.4–1.3)
Former/current	2 (8.7)	21 (91.3)	1.2 (0.3–5.3)
			
*Oral contraceptive use*			
Never/never (referent)	106 (5.1)	1970 (94.9)	1.0
Never/current	10 (10.6)	84 (89.4)	1.3 (0.6–2.6)
Current/current	41 (7.5)	505 (92.5)	1.0 (0.7–1.6)
Current/former	51 (6.9)	684 (93.1)	1.0 (0.7–1.5)
Former/former	139 (5.6)	2342 (94.4)	0.9 (0.7–1.2)
Former/current	22 (8.6)	233 (91.4)	1.1 (0.7–1.9)
			
*HPV-16 status*			
Negative/negative	316 (5.4)	5516 (94.6)	1.0
Positive/negative	14 (16.3)	72 (83.7)	3.0 (1.6–5.5)
Negative/positive	31 (30.1)	72 (69.9)	6.4 (4.0–10.2)
Positive/positive	11 (37.9)	18 (62.1)	10.0 (4.5–22.2)

a Numbers in cells do not always equal total number due to missing values.

). It is important to note that when stratified by lifetime number of sexual partners, one or more sexual partners during study follow-up remained associated with risk for seroconversion; likewise, when stratified by number of sexual partners during follow-up, two or more lifetime number of sexual partners reported at enrolment also remained significantly associated with risk for seroconversion. Compared to never smokers, a 2.8-fold increase in risk is observed for women who were smokers at enrolment and former smokers at the time of follow-up; no other category of smoking increased risk for seroconversion. Finally, women HPV-16 DNA positive at enrolment and at follow-up possessed the highest level of risk for HPV-16 seroconversion with an odds ratio of 10.0 (95% CI: 4.5–22.2), followed by a 6.4-fold risk increase for women who were initially HPV DNA negative but positive at follow-up, and a three-fold risk increase for women who were HPV DNA positive at enrolment and negative at follow-up; all were statistically significant.

### Determinants of HPV-16 seropersistence

Univariate analyses of women with serologic persistence, compared to women who were seronegative at follow-up, demonstrated recent number of sexual partners and former OC use as possible risk factors for seropersistence. After adjustment for age and serology batch, and excluding women who remained virgins for the entire study, multivariate analyses revealed that women reporting one sexual partner during follow-up and those reporting former OC use at both enrolment and follow-up had a 1.5-fold statistically significant increase in risk for serologic persistence (Table 3

**Table 3:** Final logistic regression model demonstrating the association between number of sexual partners during follow-up, former oral contraceptive use, and HPV-16 DNA status for HPV-16 seropersistence in the Guanacaste Study (*n*=1205), adjusting for age and serology batch, and excluding virgins

**Determinants of HPV-16 seropersistence**	**HPV-16 seropersistence (*n*=672)^a^ (no. (%))**	**HPV-16 seroclearance (*n*=533)^a^ (no. (%))**	**OR (95% CI)**
*Lifetime number of sexual partners at enrolment*			
0–1 (referent)	236 (53.3)	207 (46.7)	1.0
2–3	236 (56.1)	185 (43.9)	1.1 (0.8–1.4)
4+	87 (57.6)	64 (42.4)	1.0 (0.7–1.5)
			
*Number of sexual partners during follow-up*			
0 (referent)	377 (53.6)	326 (46.4)	1.0
1	122 (62.9)	72 (37.1)	1.5 (1.0–2.1)
2+	56 (49.6)	57 (50.4)	0.8 (0.5–1.3)
			
*Smoking*			
Never/never (referent)	548 (54.7)	454 (45.3)	1.0
Never/current	11 (73.3)	4 (26.4)	2.7 (0.9–8.9)
Current/current	31 (62.0)	19 (38.0)	1.4 (0.8–2.6)
Current/former	21 (58.3)	15 (41.7)	1.3 (0.7–2.7)
Former/former	45 (59.2)	31 (40.8)	1.3 (0.8–2.1)
Former/current	7 (70.0)	3 (30.0)	1.6 (0.4–6.6)
			
*Oral contraceptive use*			
Never/never (referent)	181 (51.4)	171 (48.6)	1.0
Never/current	7 (58.3)	5 (41.7)	0.9 (0.2–3.0)
Current/current	56 (47.9)	61 (52.1)	0.8 (0.5–1.3)
Current/former	86 (54.0)	73 (46.0)	1.0 (0.7–1.6)
Former/former	309 (61.3)	195 (38.7)	1.5 (1.1–2.1)
Former/current	21 (55.3)	17 (44.7)	1.2 (0.6–2.4)
			
*HPV-16 status*			
Negative/negative	583 (54.7)	482 (45.3)	1.0
Positive/negative	36 (70.6)	15 (29.4)	2.2 (1.2–4.1)
Negative/positive	7 (63.6)	4 (36.4)	1.2 (0.3–4.3)
Positive/positive	22 (78.6)	6 (21.4)	3.3 (1.3–8.4)

a Numbers in cells do not always equal total number due to missing values.

). In addition, women HPV-16 DNA positive at both enrolment and follow-up had a 3.3-fold increase in risk for HPV-16 seropersistence followed by a 2.2-fold increase in risk for women HPV-16 DNA positive at enrolment but negative at follow-up. Further stratification by age (<30 and ⩾30 years) did not alter these results.

### HPV-16 viral load and OD measurements

Increasing HPV-16 viral load at enrolment as measured by qualitative PCR signal strength (weak *vs* strong, as assessed by two observers with adjudication in case of disagreement) was also associated with a significant higher odds ratio for HPV-16 seroconversion. Odds ratios were 3.0 (95% CI: 1.5–6.0) and 6.2 (95% CI: 3.5–11.1) for weak and strong HPV-16 PCR signal strengths, respectively. Viral load did not appear to modify the risk of seropersistence among HPV-16-positive women, with odds ratios of 2.7 (95% CI: 1.2–6.4) and 2.2 (95% CI: 1.1–4.2) for weak and strong signal strengths, respectively. Further stratification by age also did not reveal differences. Further investigation of determinants of serologic persistence also revealed that of women seropositive at enrolment (e.g. five standard deviations above the set cut-point), women with the highest mean OD measures at enrolment were most likely to remain seropositive at follow-up, as defined by the same cut-point. Specifically, 80% of women with the highest quartile of mean OD seropersisted; these women possessed a 12.7-fold increase in risk for seropersistence when compared to seropositive women in the lowest quartile. In all, 5.8- and 2.0-fold increases in risk for seropersistence were observed for women in the third and second quartiles of mean OD, respectively. In the third quartile of mean OD, 69% seropersisted, and in the second quartile 44% seropersisted.

### Association between serologic status and CIN3/cancer

Finally, we observed significant associations between serologic status and disease outcome of CIN3/cancer diagnosed during follow-up as shown in Table 4

**Table 4:** Association between HPV-16 seroconversion, seroclearance, and seropersistence with development of CIN3/cancer during follow-up

**HPV-16 serological status**	**Enrolment**	**Follow-up^a^**	**CIN3/cancer**		
			**Total *n*^b^**	***n***	**OR (95% CI)**
Negative	−	−	5765	42 (0.7%)	1.0 (referent)
Clearance	+	−	501	5 (1.0%)	1.4 (0.5–3.5)
Conversion	−	+	384	12 (3.1%)	4.4 (2.3–8.4)
Persistence	+	+	631	16 (2.5%)	3.6 (2.0–6.3)

a Follow-up serology measurement is concurrent with time of disease diagnosis.

b Does not equal 7986 due to missing outcome variables.

. Compared to women who were HPV-16 seronegative at both time points, women who seropersisted or seroconverted were at elevated risk for CIN3/cancer. Women who became seronegative at follow-up were not at significant increase in risk for CIN3/cancer. When restricted to HPV-16 DNA-positive women as measured by PCR, risk associations between serology and disease were no longer statistically significant.

## DISCUSSION

In the present study, serologic status was a dynamic result of seroconversion and clearance; only half of all seropositive women were seropositive at both time points. In our previous cross-sectional study, seroprevalence peaked at 35–44 years old (Wang *et al*, 2003); in the present analysis, seropersistence was also highest in this age group. Seroconversion was highest in women 18–24 years old and steadily declined with age. This pattern mirrored HPV DNA age curves we reported previously, with a peak at youngest age groups and declining with age (Wang *et al*, 2003). Although of interest, detailed assessment of HPV DNA and serological status particularly in older age groups could not be assessed due to small numbers in these older age groups. Nevertheless, age-specific patterns of HPV-16 serologic conversion and persistence support the known waning of serological response with age and time.

In women identified as HPV-16 DNA positive at enrolment, less than half were HPV-16 seropositive at enrolment, consistent with previous studies (Carter *et al*, 2000). Although an additional 13% of seronegative women at enrolment were subsequently seropositive at follow-up, this could be the result of either HPV-16 infection identified at enrolment or from a subsequent infection (e.g. with the same or different HPV-16 variant).

Determinants of seroconversion at follow-up were similar to the determinants of seroprevalence we reported previously in our cross-sectional analysis (Wang *et al*, 2003), which were generally consistent with other populations (Stone *et al*, 2002; Nonnenmacher *et al*, 2003); recent and lifetime number of sexual partners and smoking behaviour remained associated with seroconversion. Curiously, increased risk appeared to be especially elevated among women who were current smokers at enrolment but former smokers at the time of follow-up, potentially suggesting temporality related to smoking behaviour, its concurrence with HPV exposure, and subsequent detection of antibodies. As expected, the highest risk for seroconversion was observed for women HPV-16 DNA positive both at enrolment and at follow-up. Women HPV-16 DNA positive at enrolment but negative at follow-up exhibited the next highest level of risk for seroconversion, supporting the notion that HPV antibody detection is a marker of past infection.

Determinants for seropersistence included having one sexual partner during the follow-up period and former OC use. For reasons that are not clear, having two partners during follow-up was not associated with seropersistence; continued HPV exposure (and possibly re-exposure) with the same HPV type and resultant antigenic stimulation is to be investigated in a more refined analysis of HPV DNA typing for all years of follow-up. We also found that for seropositive women, a higher OD at enrolment was associated with seropersistence at follow-up, consistent with a previous study in which HPV antibodies persisted when initial levels were high or when there was continued exposure (Ho *et al*, 2004). It is unclear why former OC use was associated with increased risk for seropersistence. Although former OC users were older, our analysis was adjusted for age, and separate analyses stratified by age did not demonstrate differences; former OC use was not related to mean OD levels.

Although IgG seroconversion against HPV-16 has been reported within 6–12 months of infection (Wikstrom *et al*, 1995; Carter *et al*, 2000), the duration of serologic detection (e.g. being seropositive) remains unclear (Stone *et al*, 2002). Reported to be high over a long period of time (van Doornum *et al*, 1998), serology measures are likely to be confounded by repeated exposure to HPV-16 (Onda *et al*, 2003). Persistence of HPV-16 antibodies has been found for 7–13 years (Shah *et al*, 1997) and for 4 years in a study of 1656 pregnant women who demonstrated stable antibody levels (af Geijersstam *et al*, 1998). We defined seropersistence as two high positive antibody measurements at enrolment and at follow-up, that is, for a median duration of over 6 years. Because serology was only assessed at two time points, we could not assess the duration of the serologic response or its determinants. Further investigation of multiple, longitudinal measurements of HPV infection assessed by both serology and DNA testing during the 5–7 years of follow-up is likely to provide additional clues regarding seropersistence.

Several studies have reported a 2–3-fold magnitude of risk for cervical cancer for HPV seropositive women, compared to seronegative women (Nonnenmacher *et al*, 1995, 1996; Chua *et al*, 1996; Wideroff *et al*, 1996; Dillner *et al*, 1997; Sigstad *et al*, 2002). In this study, we found that seropositive women who are seronegative by follow-up are not at increased risk for CIN3/cancer. On the contrary, women who seroconvert or are seropersistent possessed the highest risk for CIN3/cancer. However, the lack of association when restricted to HPV-16 DNA-positive women suggests the overwhelming association between HPV DNA and disease.

Limitations of our study include the necessity for a high cutoff (five standard deviations above mean for studied virgins) to define HPV-16 seropositive women. Because VLP ELISAs are still prone to misclassification including false positives, we chose to define HPV-16 seropositive women stringently, thus probably misclassifying some HPV-16 seropositive women (seroconverters or seropersisters) as seronegative and biasing our results towards the null. However, we attempted to measure the accuracy of our definition of seropersistence by a small subset of approximately 1500 women, and measurements were conducted 1 year after enrolment. While the majority of women defined as seropersistent (based on enrolment and 5–7 years of follow-up) were also seropositive at 1 year after enrolment, approximately 10% were not. This probably reflects misclassification in our definition of seropersistence and might also have further biased our results towards the null.

To our knowledge, this is the largest population-based seroprevalence study of HPV-16 with follow-up serology data. Our subjects were representative of the adult female population of Guanacaste, Costa Rica and our results provide a detailed description of serologic status in this and population behavioural factors likely to influence long-term serologic status. The known waning of HPV antibody detection with age is confirmed, attributed to both decline in seroconversion and seropersistence; these age-specific data may be relevant to future plans for HPV vaccination. Because antibody titres after natural infection are lower than those following vaccination, these immunologic responses should be distinguishable. Future analysis assessing longitudinal HPV DNA status in relation to serologic status will be important for clarifying the role that transient and persistent HPV infections play in initiating and sustaining a humoral immune response.
